# New Perspectives on the Role of *α*- and *β*-Amylases in Transient Starch Synthesis

**DOI:** 10.1371/journal.pone.0100498

**Published:** 2014-06-27

**Authors:** Alex Chi Wu, Jean-Philippe Ral, Matthew K. Morell, Robert G. Gilbert

**Affiliations:** 1 Tongji School of Pharmacy, Huazhong University of Science and Technology, Wuhan, Hubei province, China; 2 Centre for Nutrition and Food Sciences, Queensland Alliance for Agricultural and Food Innovation, The University of Queensland, Brisbane, Queensland, Australia; 3 Food Futures National Research Flagship, CSIRO, Canberra, Australian Capital Territory, Australia; 4 Plant Industry, CSIRO, Canberra, Australian Capital Territory, Australia; University of Insubria, Italy

## Abstract

Transient starch in leaves is synthesized by various biosynthetic enzymes in the chloroplasts during the light period. This paper presents the first mathematical model for the (bio)synthesis of the chain-length distribution (CLD) of transient starch to aid the understanding of this synthesis. The model expresses the rate of change of the CLD in terms of the actions of the enzymes involved. Using this to simulate the experimental CLD with different enzyme combinations is a new means to test for enzymes that are significant to the rate of change of the CLD during synthesis. Comparison between the simulated CLD from different enzyme combinations and the experimental CLD in the leaves of the model plant *Arabidopsis thaliana* indicate *α*-amylase, in addition to the core starch biosynthetic enzymes, is also involved in the modification of glucans for the synthesis of insoluble starch granules. The simulations suggest involvement of *β*-amylase, in the absence of *α*-amylase in mutants, slows the rate of attaining a crystalline-competent CLD for crystallization of glucans to form insoluble starch. This suggests a minor role of *β*-amylase in shaping normal starch synthesis. The model simulation predicts that debranching of glucans is an efficient mechanism for the attainment of crystalline-competent CLD; however, attaining this is still possible, albeit slower, through combinations of *α*- and *β*-amylase in the absence of isoamylase-type debranching enzyme. In *Arabidopsis* defective in one of the isoamylase-type debranching enzymes, the impact of *α*-amylase in starch synthesis is reduced, while *β*-amylase becomes significantly involved, slowing the rate of synthesis in this mutant. Modeling of transient starch CLD brings to light previously unrecognized but significant effects of *α*- and *β*-amylase on the rate of transient starch synthesis.

## Introduction

Starch in the photosynthetic tissues of plants such as leaves serves as one of the principal sinks for carbon fixed during the day and is degraded during the subsequent night to provide a continued supply of sugars to sustain respiration and growth. Starch is solely made up of glucose residues linked by *α*-(1→4) and *α*-(1→6) glycosidic linkages. It is composed of two main fractions: amylose, mostly linear with few long-chain branches, represents between 20 to 30% of the polysaccharide dry mass; amylopectin is the main component and is highly branched, with around 5% *α*-(1→6) branch points [1].

Transient starch biosynthesis in leaves requires the concerted actions of various types and isoforms of starch biosynthetic enzymes [2], [3]. The synthetic pathway is generally similar to that in other plants [2]. This first involves a supply of soluble ADP-glucose synthesized by the ADP-glucose pyrophosphorylase. This serves as the substrate for starch synthases (SSs), which catalyze the elongation of glucan chains of *α*-(1→4) linked glucose residues from the non-reducing end. There are two groups of SSs in plants: granule-bound SSI (GBSSI) and the soluble SSs. The soluble SSs are principally involved in elongation of amylopectin branches while GBSSI is mainly involved in amylose biosynthesis. Starch branching enzymes (SBEs) are responsible for generating new amylopectin branches via *α*-(1→6) branch points, by cleaving an *α*-(1→4) linkage on a donor chain and transferring the donor segment to a new *α*-(1→6) position, forming a new branch. There are minimum chain length requirements (constraints) on the number of glucose units on both the donor and the residual segments for branching to proceed (e.g. [4], [5]); these minimum number of glucose units are denoted by *X*_min_ and *X*_0_, respectively [6]. There are two groups of debranching enzymes (DBEs): isoamylase-type (ISA) including three different isoforms ISA1, ISA 2 and ISA3 respectively; and pullulanase-type (PUL) DBEs [7], [8]. Both types catalyze the hydrolysis of *α*-(1→6) linkages, releasing linear or branched glucans [2], [9]. It has been proposed that debranching of glucans by the ISA-type DBE is required for trimming of improperly positioned branches, which would otherwise delay (or prevent) crystallization. In *Arabidopsis*, both *AtISA1* and *AtISA2* genes are required to produce the heteromeric multsubunit complex, named Iso1, which contribute to the major isoamylase activity [7], [8]. Both Myers *et al*. [1] and Delatte *et al*. [8] proposed that glucans that do not crystallize efficiently are susceptible to a continued enzymatic modifications, leading to accumulation of phytoglycogen. Lack of the ISA-type DBE does not generally abolish amylopectin synthesis completely. The amylopectin formed under this circumstance has more short chains with a degree of polymerization (DP) *X*<8–12, depending on the species (e.g. [7], [8], [10]). The role of PUL-type DBE is more specific. Under normal conditions, the PUL-type DBE acts on pullulan, maltotriose units linked by *α*-(1→6) linkages, and only accounts for a minor role in transient starch synthesis [7]. In *Arabidopsis* ISA2 and PUL-type DBE double mutant *Atisa2*/*Atpu1*, starch content is further reduced compared to that in just the absence of Iso1 in mutants defective in *AtISA1* or *AtISA2*, which suggests some partial redundancy between the two DBE types [7].

While the above enzymes are the key actors essential for starch synthesis, hypotheses have emerged that other types of enzymes could contribute to transient starch synthesis under specific circumstances. For example, in the absence of all DBEs, glucans are susceptible to *α*- and *β*-amylolysis [11]. *α*-amylase (AMY) (EC 3.2.1.1) is an endoamylase hydrolyzing *α*-(1→4) linkages to release soluble glucans, either linear or branched. While AMY is known to be the main enzyme involved in the degradation of storage starch granules during cereal seed germination, its role may differ in transient starch degradation in leaves [12]. Three isoforms of AMY have been described in *Arabidopsis* with only one chloroplasticly localized AMY3 (AtAMY3) [13]. Mutants for each of the AMY or combinations do not show any alternation in the diurnal pattern of starch turnover [13], which could suggest that there is no involvement of AMY during the transitory starch synthesis or degradation phases. However, recent work from Streb *et al*. [14] overruled that postulate, suggesting a key role of AtAMY3 in starch catabolism. Very recently, Seung *et al*. [15] have described a light-dependent redox activation of AtAMY3, suggesting a counterintuitive activation of this endoamylase during starch synthesis.

An exoamylase, *β*-amylase (BAM), hydrolyzes every second *α*-(1→4) linkage from the non-reducing end to release maltose. BAM stops hydrolysis 2–4 glucose residues ahead of a branch point on the glucan branches generated by the branch points, or on those that carry other branch points. It is the major enzyme for transient starch granule breakdown in leaves. Lack of BAM leads to a starch-excess phenotype in the leaves of potato and *Arabidopsis* which is caused by a slow rate of degradation of starch over the dark period [16], [17]. Phosphorylation of transient starch plays a crucial role in initiating degradation of the granule [18]. Two sets of genes, Glucan-Water-Dikinase (GWD) and Phospho-Glucan-Water-Dikinase (PWD), have been shown to phosphorylate starch in plants [19], [20], both catalyzing the transfer of the *β*-phosphate from ATP to a glucosyl residue in the C-6 and C-3 position, respectively [21]. The limit-Dextrinase (Disproportionation) enzyme, or D-enzyme, (DPE) (EC 2.4.1.25) is also present in *Arabidopsis* chloroplasts and has been described as being involved in starch metabolism in *Chlamydomonas reinhardtii* [22], [23]. However, the *Arabidopsis* DPE mutant accumulates starch at a normal rate [24] and therefore its involvement in transient starch synthesis in *Arabidopsis* remains unclear. Finally, it was shown in *Chlamydomonas* and rice that starch phosphorylase (SP) (E.C 2.4.1.1) is involved in storage starch synthesis [25], [26]. Whether SP has the same role in leaf transient starch synthesis is currently not known [27].

**Figure 1:**
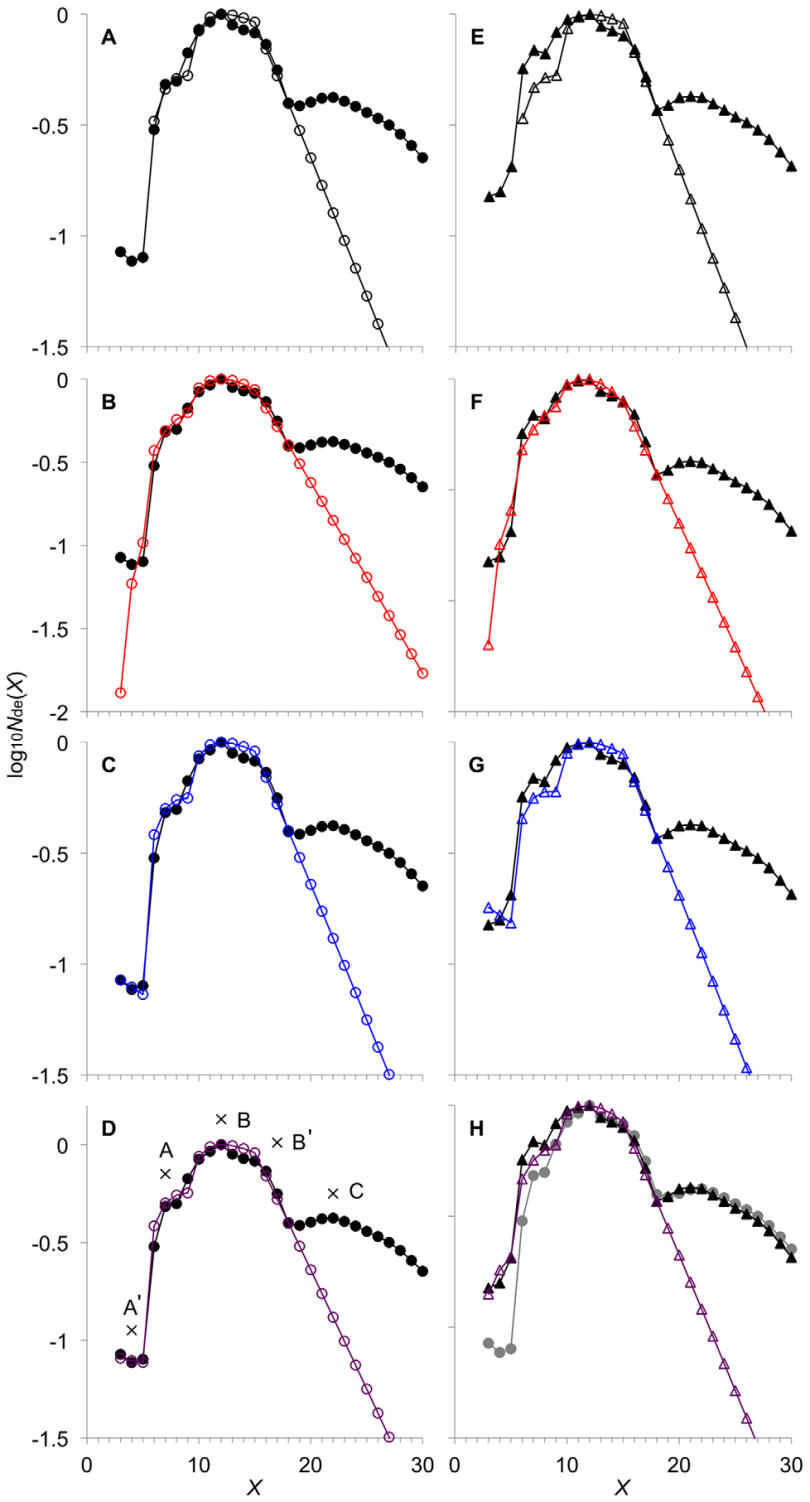
*Arabidopsis* leaf starch CLD fitted to different combinations of AMY and BAM. *N*_de_(*X*) (arbitrary units) from the wild type WT CLD (black filled circles) and the ISA-type DBE mutant defective in the *AtISA1* gene *Atisa1-1* CLD (black filled triangles) are replotted from ref. [34]. Crosses and letters indicate features in the CLD. Note the letters in bold font indicate the panels, and are not part of the CLD features. (**A**) and (**E**): simulated CLD with AMY^–^/BAM^–^ fitted to WT CLD and *Atisa1-1* CLD, respectively; (**B**) and (**F**): that for AMY^–^/BAM^+^; (**C**) and (**G**): that for AMY^+^/BAM^–^; (**D**) and (**H**): that for AMY^+^/BAM^+^. Simulated CLD are shown by different colored shapes corresponding to the different combinations of AMY and BAM. Fitting is to the enzyme set (i) range (3≤*X*≤18). (**B**) and (**D**) are compressed on the ordinate. The gray filled circles represent WT CLD replotted for comparison with *Atisa1-1* CLD.

The starch chain-length distribution (CLD) is the relative number distribution *N*_de_(*X*) of glucan chains with DP of *X* (the subscript “de” is used because these glucan chains are obtained from isoamylase-type debranching enzyme digestion of the starch). The quantity *X* = 1,2,3, … is a discrete variable. To avoid proliferation of subscripts, we use the notation *N*_de_(*X*) on the understanding that *X* only takes integer values There are several features in the CLD, for example as shown in Figure 2 of ref. [28]. These features are ascribed to the enzymatic actions involved in the modification of glucans which then crystallize to form insoluble starch granules. They have been described for starch in cereal endosperms with multiple enzyme sets [6], [28], denoted (i), (ii) … below. *Arabidopsis* starch CLD also exhibits several features (Figure 1D).

**Figure 2:**
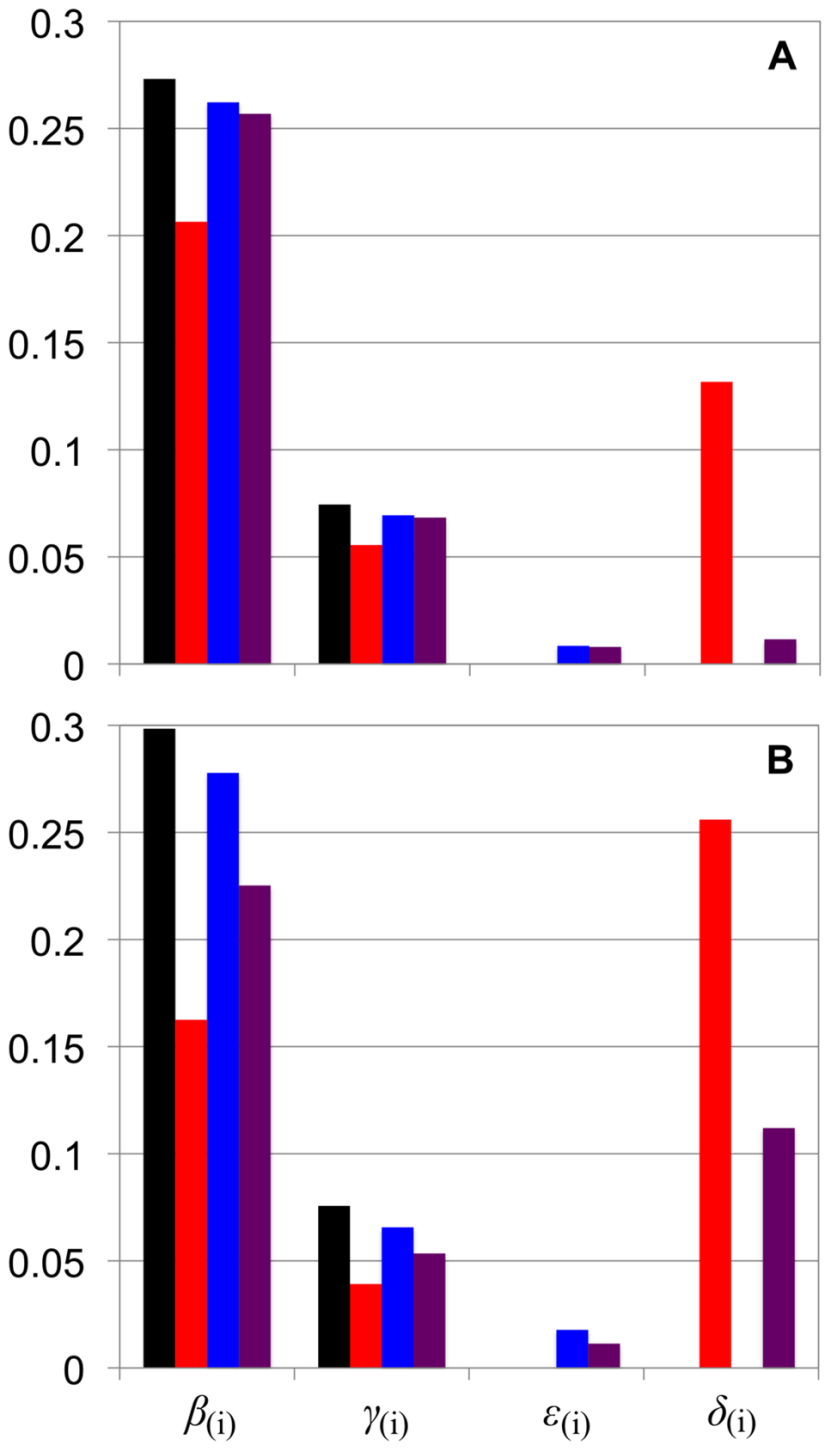
Enzymatic rate ratios from fitting *Arabidopsis* leaf starch CLDs in Figure 1. The different color bars match those used for the different combinations of AMY and BAM in Figure 1: AMY^–^/BAM^–^ (black); AMY^–^/BAM^+^ (red); AMY^+^/BAM^–^ (blue); AMY^+^/BAM^+^ (purple). (**A**): Fitted enzymatic rate ratios for WT CLD; (**B**): that for *Atisa1-1* CLD. Values of *X*_0(i)_ and *X*_min(i)_ of 6 and 10, respectively, were used for all fits.

This paper presents the first mathematical model to describe transient starch CLD (e.g. in *Arabidopsis*) by considering the actions of the biosynthetic enzymes involved. This new modeling would appear to provide an innovative and powerful option for identifying and testing candidate enzymes that have not previously been considered to be involved in starch biosynthesis. Comparison between experimental data for *Arabidopsis* and the model simulation suggests that, in addition to the core starch biosynthetic enzymes, *α*-amylase is also involved in the synthesis of insoluble starch granules. In the absence of Iso1, *β*-amylase becomes significantly involved. Furthermore, modeling of transient starch CLD reveals an unexpected role of *α*- and *β*-amylase in transient starch synthesis in *Arabidopsis*.

## Mathematical Modeling

### Leaf starch CLD model

The model expresses the rate of change of the glucan CLD at time *t*, *∂N*_de_(*X*)/*∂t*, in terms of the actions of the enzymes that are assumed to be involved in the modification of glucans which then crystallize and deposit to form insoluble starch granules. Whether or not an enzyme is considered to be involved for the purpose of this model is defined by whether or not the corresponding mutant possesses an altered starch CLD. The following discusses the evidence for the enzymes considered in the model and the reasons why others are not. The properties of enzymes described in the following are based on their general properties and not species specific. This allows a general mathematical model of starch CLD to be developed and is then used to compare with experimental data for *Arabidopsis*. The results, which will be specific to *Arabidopsis*, will be seen to provide further understanding of starch biosynthesis in *Arabidopsis* and perhaps more generally.

(1) SS, SBE and the DBEs, the core enzymes, undoubtedly contribute to *∂N*_de_(*X*)/*∂t* during the synthetic phase. Mutations in all of the three enzyme types show changes in the starch CLD (e.g. [7], [29], [30]). Storage starch synthesis is modeled by the three enzyme types [6], [28].

(2) There is no indication whether *α*-amylase contributes to *∂N*_de_(*X*)/*∂t* during the synthetic phase. *Arabidopsis* AtAMY3 knock-out mutant has been generated [13], but the mutant CLD was not reported. Therefore, we cannot discount the possibility of *α*-amylase contributing to *∂N*_de_(*X*)/*∂t*. The exact contribution of AMY to *∂N*_de_(*X*)/*∂t*, however, is a complex one. Just to give a few examples, AMY is modulated by the presence and clustering of *α*-(1→6) branch points and higher structures (e.g. by the distance between clusterings, and by glucan conformation such as the formation of *α-*helices between adjacent branches). As there are a vast number of structural possibilities for the substrates, it is currently impossible to determine the exact substrate specificity of AMY over the entire course of starch biosynthesis. At the very least, the time evolution of *α*-(1→6) linkage positions would need to be considered during starch synthesis, which would give information on the position of the branches, to partly capture the contribution of AMY to *∂N*_de_(*X*)/*∂t*. However, this information is not currently available. With such uncertainty, it is best to consider, in this first approach, only the most probable mechanism of AMY towards starch biosynthesis. As stated, glucans that are yet to crystallize represent the newly formed layer of material on starch molecules and as such are most susceptible to enzymatic actions. It has been shown that these materials comprise mainly short A-chains (DP 6–11) [4]. Therefore, we assumed that, during the synthesis of glucans, when a chain is hydrolyzed by AMY, only the *α*-(1→6) linkage that carries the branch exerts effects on the enzyme. Refinement of this assumption may be necessary for the long B-chains and beyond where there are higher structural features which can influence the contribution of AMY. For example, clusters of branch points hinder AMY, so AMY would preferentially hydrolyze *α*-(1→4) linkages away from the clusters.

(3) *Arabidopsis β*-amylase mutants have been generated by Fulton *et al*. [16], but the mutant CLD was not reported. The presence and clustering of *α*-(1→6) branch points and phosphate residues on glucan branches block BAM [31], [32]. There is currently no information on the time evolution of *α*-(1→6) linkage and phosphate residues positions. Therefore, the exact contribution of BAM to *∂N*_de_(*X*)/*∂t* cannot be established. As with *α*-amylase, we assume that when a branch is hydrolyzed by BAM, only the *α*-(1→6) linkage that carries the branch has an effect on *β*-amylolysis.

(4) There is no indication that GWD and PWD participate directly in the synthesis or hydrolysis of *α*-(1→4) or *α*-(1→6) linkages. Therefore we can conclude that GWD and PWD do not contribute to *∂N*_de_(*X*)/*∂t* directly. An indirect effect is possible if BAM is involved in the synthesis of transient starch. In which case, its exoamylasic activity can be blocked by the phosphate residues which come from phosphorylation by GWD and PWD. This could, however, be considered as marginal, as typically only one in 2000 glucose residues is phosphorylated (e.g. *Arabidopsis* leaf starch at the end of the synthetic phase) [3]. We can thus expect only a minute amount of phosphate residues on an average branch. Therefore, we assume that neither GWD nor PWD contributes significantly to *∂N*_de_(*X*)/*∂t*. In addition, a mutation in the GWD encoding gene that leads to amylopectin with no phosphorylation in *Arabidopsis* gives no significant difference in the amylopectin CLD [33].

(5) Critchley *et al*. [24] showed that there is no change in the starch CLD of *Arabidopsis* DPE mutant. Therefore, we conclude that DPE does not contribute to *∂N*_de_(*X*)/*∂t*. This contrasts with *Chlamydomonas reinhardtii*, where DPE mutant exhibits an increase in *X* = 3–5 [22], [23]. This difference suggests a different requirement for starch biosynthesis in unicellular green algae compared to that in higher plants.

(6) In *Chlamydomonas*, mutation in one of the three SP affects the starch CLD by causing a reduction in *X* = 6–13. Whether SP has the same effect in *Arabidopsis* is unknown. In theory, the contribution of SP to *∂N*_de_(*X*)/*∂t* is either the same or opposite of SS, depending on whether SP polymerizes or depolymerizes glucan chains, respectively. Therefore, we assumed that it either adds or counters the rate of glucan elongation, respectively. This simply means the predicted rate of glucan elongation would be a combined rate of SS and SP. The overall rate, however, would have to be a positive contribution to propagation, or otherwise starch synthesis would not be possible.

Considering the above, the synthesis of the transient starch CLD in leaves is modeled by formulating *∂N*_de_(*X*)/*∂t* with respect to the contributions of SS, SBE, DBE, AMY and BAM. Note that DBE(i) refers to both the ISA- and PUL-type DBE. They are not differentiated in this model since both facilitate the hydrolysis of *α*-(1→6) linkages. To rigorously define the enzymatic activities of the enzymes involved we would need to consider factors such as pH change, temperature, availability of light, substrate specifies and substrate and products concentrations. One way would be to study the enzyme kinetics for the different factors and set out rate equations to describe enzymatic activities in terms of the appropriate factors. However, this would give an undesirably large number of parameters and would cause the model to always be able to be forced to agree with experiment, and would thus be irrefutable, which is scientifically disfunctional [28]. Here we assume that overall enzymatic activities, parameterized by an enzymatic rate coefficients, represent the average rate over the development of starch molecules during synthesis; with this small number of parameters, disagreement with data would indicate that the model needs modification or abandonment. Of course, the simplistic representation of the enzymatic activity can be refined, and indeed can be replaced by Michaelis-Menten equations, as explored in our previous paper [28]. In the current development, the enzymatic rate coefficient are assumed time- and chain-length independent, except for the various constraints for SBE(i), AMY(i) and BAM(i) which we can rigorously defined by Eqn 1. The evolution equation governing *∂N*_de_(*X*)/*∂t* in the enzyme set (i) range (Features A′–B′ in Figure 1D) in *Arabidopsis* leaf starch is given by Eqn 1. The right-hand side includes the contributions from SS(i), SBE(i), DBE(i), AMY(i) and BAM(i). The evolution equation governing *∂N*_de_(*X*)/*∂t* in the enzyme set (i) range (Features A′–B′ in Figure 1D) in *Arabidopsis* leaf starch is given by Eqn 1. The right-hand side includes the contributions from SS(i), SBE(i), DBE(i), AMY(i) and BAM(i).
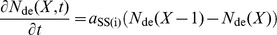





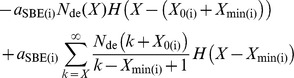





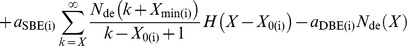





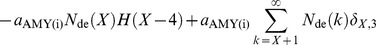





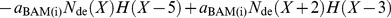
(1)


The enzymatic rate coefficient for glucan elongation by the addition of ADP-glucose by SS(i) and possibly SP(i) is *a*_SS(i)_, with units of s^–1^. *a*_SBE(i)_ is that for branching by SBE(i) in enzyme set (i), with units of *s* ^–1^; *a*_DBE(i)_, *a*_AMY(i)_ and *a*_BAM(i)_ are those for debranching, *α*- and *β*-amylolysis, respectively, all with the same units as *a*_SBE(i)_. *H*(*y*) is a step function: *H*(*y*)  = 0 for *y*<0,  = 1 for *y*≥0, and appears because of the various constraints for SBE(i), AMY(i) and BAM(i) (see Introduction and above). The Kronecker delta *δ_X_*_,3_ is 1 for *X* = 3, and zero otherwise (recall *X* is a discrete variable), and expresses that when branches are hydrolyzed by AMY, they are assumed to reduce to *X* = 3 only. *H*(*X*–3) for BAM indicates the shortest branches produced are *X* = 3 from *X* = 5, hence *H*(*X*–5) for the other BAM term in Eqn 1. The reason for *X* = 3 is because it is the shortest branch length with a significant amount in the CLD (Figure 1).

Eqn 1 can be conveniently presented in matrix form as given in Eqn 2.
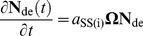
(2)


**N**_de_(*t*) is a column vector whose entries comprise *N*_de_(*X*, *t*). **Ω** is the rate coefficient matrix, whose elements comprise the rate coefficients on the right hand side of Eqn 1 divided by *a*_SS(i)_. The elements of Ω are dimensionless ratios of the enzymatic rate coefficients, denoted by *β*_(i)_  =  *a*_SBE(i)_/*a*_SS(i)_, *γ*_(i)_  =  *a*_DBE(i)_/*a*_SS(i)_, *ε*_(i)_  =  *a*_AMY(i)_/*a*_SS(i)_ and *δ*_(i)_  =  *a*_BAM(i)_/*a*_SS(i)_; these, plus *X*_0(i)_ and *X*_min(i),_ are the six model parameters for the enzyme set (i) component of the CLD.

### Time evolution of leaf starch CLD

There is evidence that during the synthesis of *Arabidopsis* leaf starch, the CLD is in a steady state [8]. A steady-state CLD, as defined previously for storage starches in cereal endosperm [6], means the relative abundance (concentration) between glucan chains of different *X* is independent of time (although the total amount of starch may change in time). Wu *et al*. [6] proposed that crystalline-competent CLD, required for the formation of insoluble starch, is in a steady state. This CLD, which describes the glucans at the periphery of growing starch granule (the soluble-insoluble interphase) crystallizes non-selectively, giving the granular CLD [28]. Modeling of the CLD of glucans at the interphase is then used to describe the granular CLD. A steady-state CLD obeys the relation *∂* **N**_de_(*t*)/*∂t*  =  **ΩN**_de_  = 0. **N**_de_(*t*), the solution of Eqn 2, a homogenous first order differential equation, is given by Eqn 3, as described elsewhere (e.g. [6]).
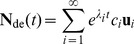
(3)




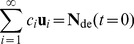
(4)


where λ*_i_* is the *i* ^th^ eigenvalue of **Ω** and **u** *_i_* is the corresponding eigenvector. Eigenvalues and eigenvectors are defined by the relation **Ωu**  =  λ**u**, where **u** is a non-zero vector. *c_i_* is given by Eqn 4, which is a system of linear equations. **N**_de_(*t = *0) is a column vector whose entries comprise *N*_de_(*X*, *t = *0), which give the CLD at the beginning of the period in question. The eigenvalues and eigenvectors of **N**_de_(*t*) for the enzyme set (i) component are controlled by the six model parameters (see above).

To attain a steady-state CLD, there must be at least one zero eigenvalue while the rest of the eigenvalues are negative. This condition ensures that **N**_de_(*t*) evolves in time to the eigenvector corresponding to the zero eigenvalue. This zero-eigenvalue condition means only a defined range of the enzymatic rate ratios (i.e. *β*_(i)_, *γ*_(i)_, *ε*_(i)_ and *δ*_(i)_) are allowed [6]. This range of rate ratios means that, in nature, there is a range of steady-state CLD. This is evident by the fact that, for example, starch CLD differs between different plant species.

A way of interpreting the time-evolution of **N**_de_(*t*) is as follows. Eqn 3 shows that the time evolution of **N**_de_(*t*) is described by the eigenvalues and the corresponding eigenvectors. When the zero-eigenvalue condition is satisfied, **N**_de_(*t*) evolves to the eigenvector corresponding to the zero eigenvalue over time. When this happens, **N**_de_(*t*) is then at a steady state. The time for this to happen depends on the values of the rest of the eigenvalues. If there are eigenvectors that correspond to less negative (close to zero) eigenvalues, they decay relatively slowly, which means a steady-state **N**_de_(*t*) will require a relatively longer time to reach. Conversely, eigenvectors that correspond to more negative eigenvalues decay rapidly.

### Fitting data for *Arabidopsis* leaf starch CLD

*Arabidopsis* leaf starch CLD within the enzyme set (i) range (Features A′–B′) from the wild-type (WT CLD) and the ISA-type DBE mutant defective in the *AtISA1* gene (*Atisa1-1* CLD) were non-linear least squares fitted to the solution of Eqn 1, **N**_de_ (Figure 1). Data were digitized from ref. [34]. The fit to the range dominated by enzyme set (i) is parameterized by the six model parameters, *β*_(i)_, *γ*_(i)_, *ε*_(i)_, *δ*_(i)_, *X*_0(i)_ and *X*_min(i)_.

## Results

### Model fitting of *Arabidopsis* leaf starch CLD

*Arabidopsis* leaf starch CLD shows a number of distinct features. Figure 1D shows the CLD replotted from data given in [34]. These features are also seen in other reports (Figure S1). We have proposed that these features are ascribed to what we denote as “enzyme sets” [28]. An enzyme set comprises those enzymes that are thought to contribute to *∂* **N**_de_(*t*)/*∂t*: for example, for storage starch in cereal endosperms, an enzyme set would be made up of one each of the various isoforms of SS, SBE and DBEs (both the ISA- and PUL-type DBE) or of these enzymes having different activity arising from their complexation state, post-translational modification, or other temporally or physically distinguished regulatory effects [28]. The enzymes in enzyme set (i) are designated SS(i), SBE(i), DBE(i). A given enzyme set often dominates some features of the CLD, but there will always be contributions from other enzyme sets in any particular features.

The features of *Arabidopsis* leaf starch CLD are as follows, with the labels chosen to be consistent with that used previously [28] and discussed extensively in that reference. Feature A′ comprises the very short chains (*X* = 3–5), which are not seen in storage starch and must be due to additional enzymatic activity. Features A and B are ascribed to enzyme set (i). Feature A is due to one of the SBE(i) chain length requirements, *X*_0(i)_, and covers a range of *X* of between *X*_0(i)_ and *X*_min(i)_. Feature B is the global maximum which is due to the other chain length requirement, *X*_min(i)_, and lies between *X*_min(i)_ and *X*_0(i)_ + *X*_min(i)_. Feature B′, not consistently observed in storage starches, is a small bump which may perhaps involves synthesis by an additional enzyme set. Feature C corresponds to the range of *Arabidopsis* leaf starch CLD beyond the “discontinuity” consistently observed at *X* = 17–18 [34]. Feature C in *Arabidopsis* leaf starch CLD is more prominent than in, for example, maize and rice endosperms (e.g. [6]), while being similar to that of the wheat endosperm. There are probably additional features after Feature C, but most *Arabidopsis* leaf starch CLD reported in the literature, obtained by high-performance anionic-exchange chromatography (HPAEC), were only given up to a range in Feature C (*X*≈30). It may be that the higher *X* range were not shown because of the limitations of HPAEC (see [35] for a review). Roldan *et al*. [36] reported *Arabidopsis* CLD with a higher *X* range (up to ∼60) when capillary electrophoresis was used instead.

The *Arabidopsis* leaf starch CLD model considers the actions of SS(i), SBE(i), DBE(i), AMY(i) and BAM(i) in enzyme set(i). When both AMY and BAM are involved in addition to the core enzymes, it is designated as AMY^+^/BAM^+^; AMY^–^/BAM^–^ means neither are involved. A combination of AMY and BAM (i.e. AMY^–^/BAM^–^, AMY^–^/BAM^+^, AMY^+^/BAM^–^ and AMY^+^/BAM^+^) were tested by fitting to the *Arabidopsis* CLD data (Figure 1). Fitted model parameters are given in Figure 2.

### The very short chains in *Arabidopsis* leaf starch CLD are due to the actions of AMY

The presence of Feature A′, the very short chains (3≤*X*≤5), in WT CLD (Figure 1D) is ascribed to the actions of AMY. Model simulation with AMY^–^/BAM^–^ fitted to WT CLD lacks Feature A′ (Figure 1A); AMY^–^/BAM^+^ gave Feature A′ but the fit is not quantitative (Figure 1B); AMY^+^/BAM^–^ and AMY^+^/BAM^+^ both gave quantitative fits to Feature A′ (Figure 1C, D). In addition, the simulated CLD is essentially the same for AMY^+^/BAM^–^ and AMY^+^/BAM^+^. The fitted model parameters between AMY^+^/BAM^–^ and AMY^+^/BAM^+^ did not show significant differences, except for a zero *δ*_(i)_ for the first case (Figure 2A). These suggest that in the wild type *Arabidopsis* AMY, but not BAM, is involved in transient starch synthesis.

*Atisa1-1* CLD was fitted with the different combinations of AMY and BAM as described above. AMY^+^/BAM^+^ give the closest fit to *Atisa1-1* CLD (Figure 1H); AMY^–^/BAM^–^, AMY^–^/BAM^+^ or AMY^+^/BAM^–^ give simulated CLDs that do not quantitatively reproduce Features A′ and A (Figure 1E, F, G, respectively). The fitted model parameters show a slight decrease in *ε*_(i)_, but a significant increase in *δ*_(i)_ with AMY^+^/BAM^+^ compared to that for AMY^+^/BAM^–^ for the wild type *Arabidopsis* (Figure 2B). This indicates the relative rate of *β*-amylolysis is significantly increased in the ISA-type DBE mutant. There is a higher BAM activity in the leaf extract of the ISA-type DBE mutant [34]. This implies that amylopectin in the DBE mutant with the elevated Features A′ and A are due to excessive *β*-amylolysis. None of the other combinations of AMY and BAM could reproduce the elevated Features A′ and A in *Atisa1-1* CLD (Figure 1E–G). This suggests that in the ISA-type DBE mutant, both AMY and BAM are involved in transient starch synthesis.

There is no significant difference in Features B and B′ between the wild-type and ISA-type DBE mutant *Arabidopsis* (Figure 1H). All fitting except with AMY^–^/BAM^–^ gave quantitative fits to the features. This further strengths the involvement of AMY in transient starch synthesis in the wild-type *Arabidopsis* and both AMY and BAM in the DBE mutant. The model can be made to encompass Feature C by an additional enzyme set. In order for the additional enzyme set to yield a prominent Feature C, such as that seen in the *Arabidopsis* leaf starch CLD (Figure 1D), the additional enzyme set is required to follow the “independent substrate model” as proposed earlier for the *Triticeae* tribe [28]. However, treatment of Feature C with the model is not deemed useful at present, because current *Arabidopsis* leaf CLD data are limited up to *X* = 30–40, which does not provide sufficient data points for accurate fitting (see above).

### Effect of AMY and BAM on rate of attainment of steady-state CLD

**Figure 3:**
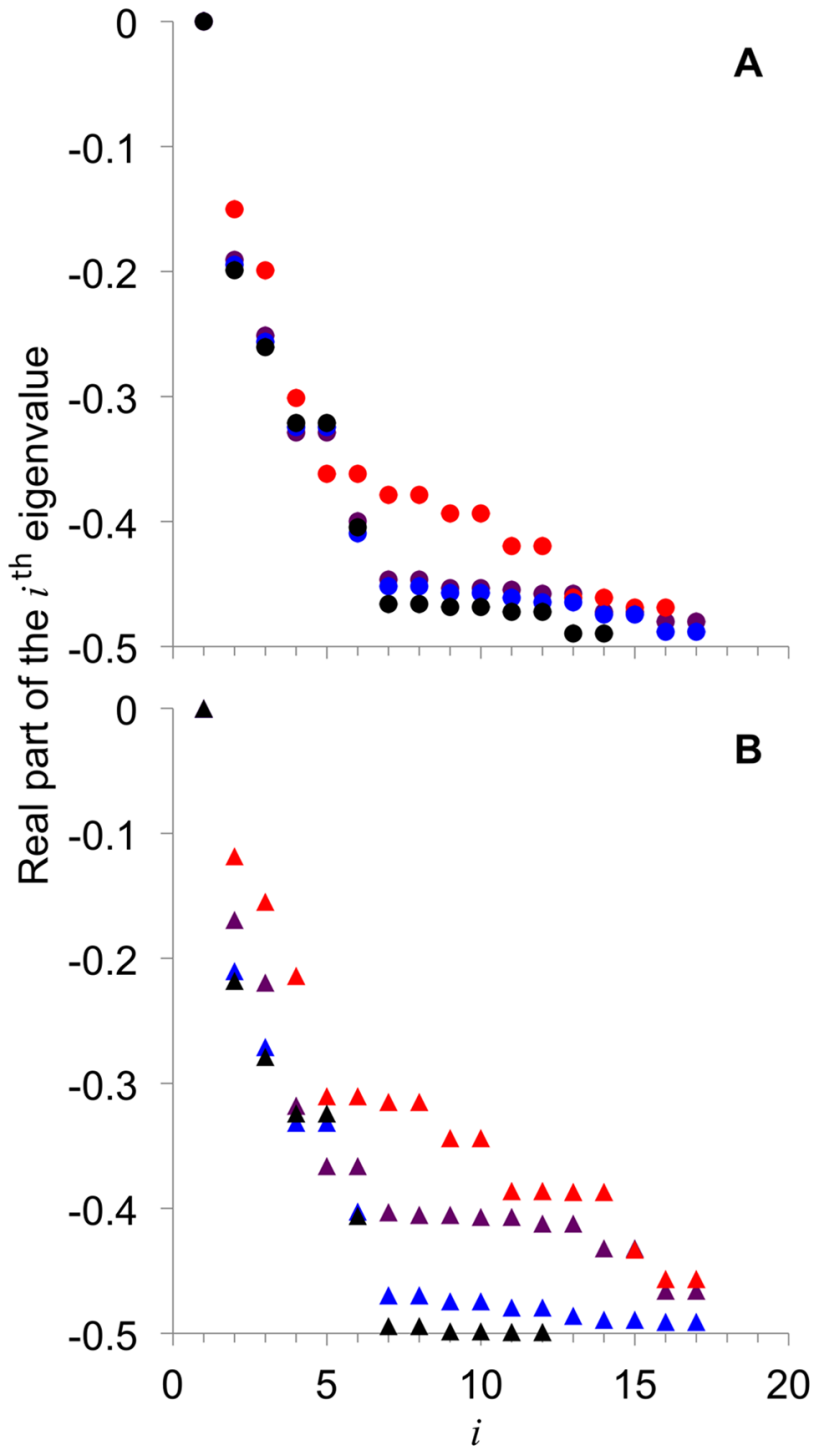
Real parts of the eigenvalues corresponding to the fitted model parameters from Figure 2. (**A**): Eigenvalues of a particular color are generated by the corresponding combination of AMY and BAM given in Figure 2 for WT CLD; (**B**): like (**A**) for *Atisa1-1* CLD. The zero eigenvalue is for *i* = 1. The higher eigenvalues are shown in Figure S2.

AMY and BAM have effects on the relative rate of steady-state CLD attainment. Figure 3 shows the eigenvalues corresponding to the fitted model parameters (Figure 2) obtained from fitting WT CLD and *Atisa1-1* CLD with the different combinations of the amylases (Figure 1). The full list of eigenvalues is given in Figure S2. There are differences in the values of the eigenvalues between the different combinations of the amylases. Only the real part of the eigenvalues is shown because the first few (largest) eigenvalues are real numbers. The largest eigenvalues have the most effect on the time-evolution of **N**_de_(*t*) (see Mathematical Modeling). The eigenvalues are either real or complex numbers with non-zero imaginary component; the imaginary components always appear in pairs with opposite sign, so the actual value of **N**_de_(*t*) is real but with some transient oscillations. It is proposed that the time evolution of the CLD before a steady state is reached is described by Eqn 3. Data for this may be observed if the CLD of the glucan at the periphery of starch granules are isolated and characterized.

For WT CLD, it is apparent that AMY^–^/BAM^+^ generated relatively small negative eigenvalues compared to AMY^+^/BAM^–^ and AMY^+^/BAM^+^ (Figure 3A). This means the relatively rate of steady-state CLD attainment is slowest in AMY^–^/BAM^+^. There are no significant differences between eigenvalues of AMY^+^/BAM^–^ and AMY^+^/BAM^+^ (Figure 3A); AMY^–^/BAM^–^ also generated similar values of the eigenvalues (Figure 3A).

For *Atisa1-1* CLD, AMY^–^/BAM^+^ also generated relatively less-negative eigenvalues (Figure 3B). There are, however, significant differences between AMY^+^/BAM^–^ and AMY^+^/BAM^+^: AMY^+^/BAM^–^ has relatively large negative eigenvalues compared to AMY^+^/BAM^+^. AMY^+^/BAM^+^ has relatively small negative eigenvalues compared to AMY^+^/BAM^–^ or AMY^+^/BAM^+^ for the wild-type plant. This indicates the relatively rate of steady-state CLD attainment in the ISA-type DBE mutant is slow compared to the wild-type plant. AMY^–^/BAM^–^ generated the largest negative eigenvalues, relatively.

There is generally a wide spread of the values of eigenvalues between the combinations of AMY and BAM for the ISA-type DBE mutant (Figure 3B), while they are more consistent in the wild-type plant, except for AMY^–^/BAM^+^ (Figure 3A).

### AMY and BAM can support the attainment of a steady-state CLD

We tested the range of the enzymatic rate ratios (i.e. *β*_(i)_, *γ*_(i)_, *ε*_(i)_ and *δ*_(i)_) that satisfy the zero-eigenvalue condition which is required to attain a steady-state CLD. In each case, the extreme case was considered: one or more of the enzymatic rate ratios is set to zero while allowing others to vary until the zero-eigenvalue condition was satisfied. *β*_(i)_  = 0 was not tested because SBEs are indispensible for amylopectin synthesis. There are eight combinations of zero and non-zero values of *γ*_(i)_, *ε*_(i)_ and *δ*_(i)_ which are shown in Table 1.

**Table 1:** Combination of zero and non-zero values of *γ*_(i)_, *ε*_(i)_ and *δ*_(i)_ in Eqn 1.

	*β*_(i)_	*γ*_(i)_	*ε*_(i)_	*δ*_(i)_
*	NZ	NZ	NZ	NZ
*	NZ	0	NZ	NZ
*	NZ	NZ	0	NZ
*	NZ	NZ	NZ	0
	NZ	0	0	0
*	NZ	0	0	NZ
*	NZ	NZ	0	0
*	NZ	0	NZ	0

NZ denotes a non-zero value for the enzymatic rate ratio; 0 denotes a zero value for the enzymatic rate ratio. * Indicates the zero-eigenvalue condition can be satisfied by the specified combination; each row is a combination.

Table 1 shows unless *γ*_(i)_, *ε*_(i)_ and *δ*_(i)_ are all zero, the zero-eigenvalue condition can be satisfied. This indicates, given SS and SBE, a steady-state CLD can be attained with any of the combinations of DBE, AMY and BAM. The combination SS, SBE and DBE has been shown in our previous paper to satisfy the zero-eigenvalue condition for storage starch [6].

## Discussion

This paper presents the first mathematical model for the synthesis of transient starch in leaves. The model considers the synergic actions of SS, SBE, DBE, the enzyme set considered to be responsible for the steady-state attainment of glucan CLD for crystallization of the amylopectin structure for insoluble starch granule formation. This work also described an innovative approach where the model also included enzymes, *α*-and *β*-amylase (AMY and BAM), that had not been previously thought to be involved in the starch biosynthesis pathway.

### Modeling suggests the involvement of AMY and BAM in transient starch synthesis in *Arabidopsis thaliana*

By comparing the simulated CLD obtained with our mathematical model to the CLD produced in normal transient starch synthesis in the wild-type *Arabidopsis*, we discovered that the core enzymes (SS, SBE, DBE) are necessary but not sufficient to produce the CLD seen in the wild-type *Arabidopsis*. Indeed, WT CLD showed an extra Feature A′ which suggested the presence of additional enzymatic activity. By including different enzymes into our mathematical model, we discovered that Feature A′ could only be predicted by a form of the model which includes the involvement of AMY. This supports our assumption that AMY mainly acts on short A-chains (DP 6–11) in the newly formed layer of material on starch molecules and not on glucans deposited in the semi-crystalline structure of starch. It is reasonable to assume that this structure is less accessible to further enzymatic modification.

From the fit by AMY^+^/BAM^–^ to the WT CLD, the model implies that AMY is responsible for the presence of Feature A′. However, further characterization using *Arabidopsis* ISA-type DBE mutant CLD suggested that both AMY and BAM were involved with BAM contributing to the elevated Feature A′ and A in the ISA-DBE mutants.

### Roles of AMY in *Arabidopsis* leaf starch synthesis

Our model suggests that, in the wild-type *Arabidopsis*, AMY is involved in starch synthesis but BAM is not. Indeed, according to our model, AMY is the enzyme responsible for the presence of Feature A′, the very short chains in the CLD. The presence or absence of BAM in our model did not influence this Feature. This suggests that AMY would act along with the ISA-type DBE to ensure the trimming of the pre-amylopectin to finalize starch crystallization.

Concerted actions of different classes of hydrolysase have been described in *Arabidopsis* transitory starch metabolism [14]. In fact, starch degradation required the simultaneous action of AtAMY3, ISA3 and D-enzyme. AtAMY3 would release branched small polysaccharides from starch granule which might be digested by the dual action of the D-enzyme and ISA3 on limit dextrin type of glucans. Although involved in the starch degradation, this mode of action could be translated into a pre-amylopectin editing system, as proposed in our model. It may be possible that structural editing by AMY is a plausible alternative and complementary pathway to the DBEs for transient starch synthesis. Despite being previously thought to be exclusively involved in starch degradation, recent work from Seung *et al*. [15] demonstrated that AtAMY3, the only chloroplastic *α*-amylase in *Arabidopsis*, is redox catalyst activated by light. Hypotheses emerged around a potential role AMY in a stress-response mechanism. However, having the unique chloroplastic AMY active during daylight could also imply a role in the synthetic phase.

**Figure 4:**
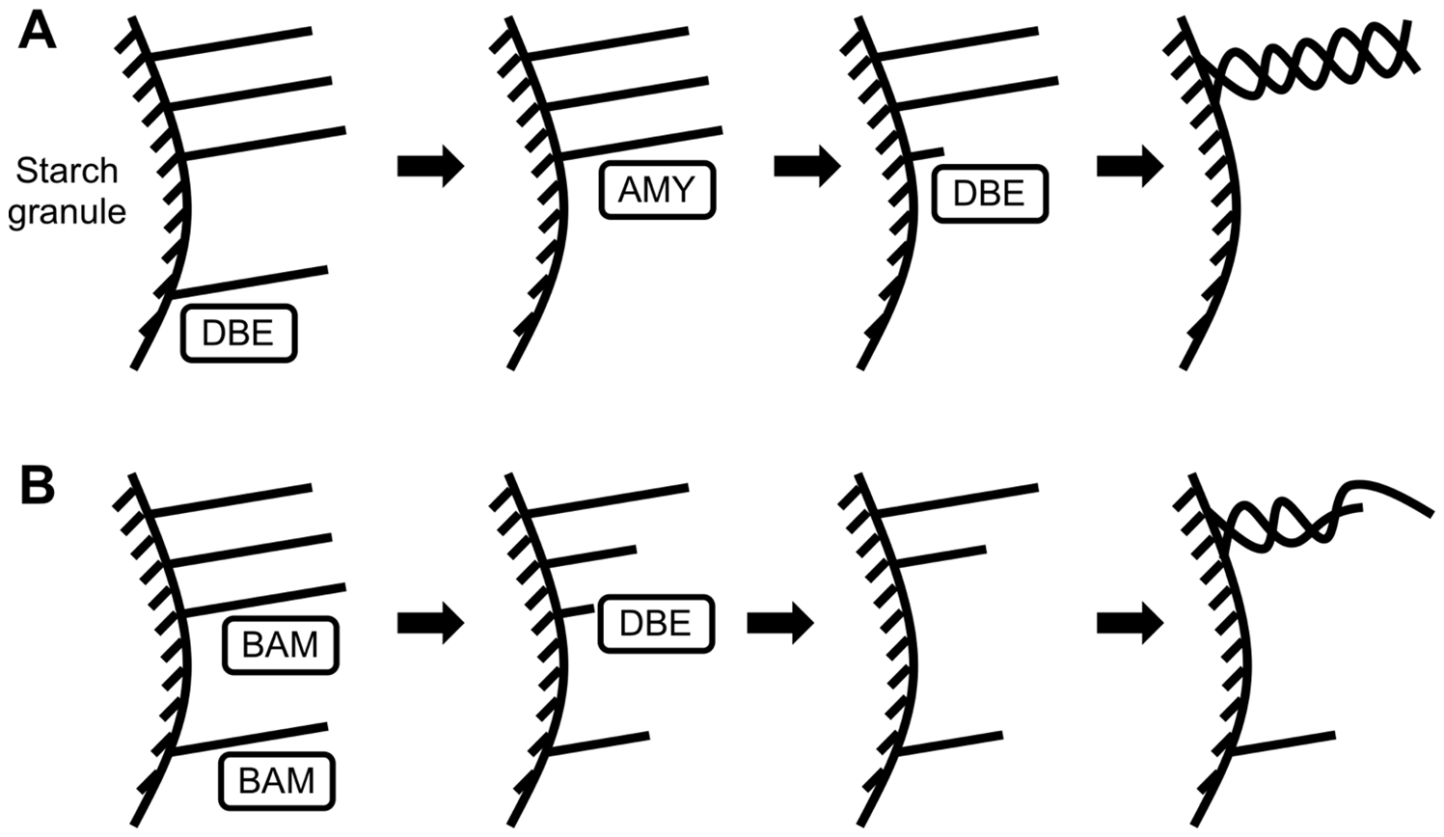
Model for the roles of AMY and BAM in *Arabidopsis* transient starch biosynthesis. (**A**) In the wild-type plant, loosely spaced branches are trimmed by DBE effectively; AMY hydrolyzes branches to short glucan stubs for further debranching. This produces a crystallization-competent structure. (**B**) In the ISA-type DBE mutant, the highly branched polysaccharide is more susceptible to *β*-hydrolysis which produces varying lengths of glucan stubs. Debranching and trimming by AMY and residual DBE activity are ineffective and thus delay crystallization.

In the sense of pre-amylopectin trimming, *α*-amylolysis can be seen as a complementary pathway. Indeed, AMY has the potential to hydrolyze *α*-(1→4) linkages further away from the non-reducing ends of glucan branches, thereby yielding shorter glucan stubs. These shorter glucan stubs may become susceptible to DBEs (Figure 4A). This is more efficient than *β*-amylolysis, as hydrolysis by BAM occurs only every second *α*-(1→4) linkage from the non-reducing ends. When BAM act on the highly branched polysaccharide produced in the ISA-type DBE mutant, it produces glucan stubs varying in lengths, which may not be the optimal substrate for debranching and may delay crystallization (Figure 4B). This is supported by our result showing that AMY^–^/BAM^+^ results in a slower rate of steady-state CLD attainment.

Furthermore, in the ISA-type DBE mutant *Arabidopsis*, when both AMY and BAM are involved, our model shows that the relative rate at which BAM operates is considerably increased. Our model also suggests that loss of ISA-type DBE resulted in glucans more susceptible to *β*-amylolysis. This coincides with the fact that the activity of BAM is higher in the leaf extracts of the *Arabidopsis* ISA-type DBE mutant [34]. Furthermore, this agrees with an increased amount of maltose in a mutant of *Arabidopsis* lacking all DBEs [11]. This implies that part of the increase came from *β*-amylolysis of starch during the synthetic phase. On the other hand, the relative rate of *α*-amylolysis decreased. It was observed that small branched oligosaccharides were still produced in the mutant lacking all DBEs, but not in the wild-type *Arabidopsis*. This implies that in the wild-type, branched oligosaccharides are synthesized, but turned over rapidly.

### AMY and BAM influence the rate of transitory starch synthesis

The simulations suggest that the rate of steady-state CLD attainment would be the slowest with AMY^–^/BAM^+^. This suggests that involvement of BAM, but without AMY, would interfere with the trimming of the pre-amylopectin to finalize starch crystallization. This is consistent with the notion (above) that *β*-amylolysis is a less efficient mechanism for transient starch synthesis. However, the additional involvement of AMY (i.e. AMY^+^/BAM^+^) restored the rate of steady-state attainment to that of the wild-type *Arabidopsis* (AMY^+^/BAM^–^). Interestingly, *Arabidopsis* AMY knock-out mutant showed no significant change in the rate of starch accumulation in the leaves compared to the wild-type plant [13]. This is exactly what we predict with AMY^–^/BAM^–^.

In the ISA-type DBE mutant, AMY^–^/BAM^+^ is also predicted to yield the slowest rate of steady-state CLD attainment. However, the model predicts that when the involvement of BAM is removed (AMY^+^/BAM^–^), the rate of steady-state CLD attainment is comparable to that in the wild-type *Arabidopsis*. This not only suggests that BAM is detrimental to the rate of steady-state CLD attainment, but it could act preferentially on the highly branched polysaccharide produced in the ISA-type DBE mutant, reducing the efficiency of complementing enzymes, for example, the PUL-type DBE.

There is a wide spread in the eigenvalues in the ISA-type DBE mutant with the different combinations of AMY and BAM. This suggests that when the ISA-type DBE is involved, the rate of starch synthesis is insensitive to the involvement of the different combinations of AMY and BAM. This implies the ISA-type DBE is the main regulator of the rate of steady-state CLD attainment required for the formation of insoluble starch. In our model, AMY seems to have a significant but limited effect and BAM would only be detrimental to the rate of steady-state CLD attainment.

There are several inferences we can make about the roles of AMY and BAM in *Arabidopsis* transient starch synthesis from the eigenvalues. (1) A way of interpreting the zero-eigenvalue condition of the model is that steady-state CLD attainment in the wild-type *Arabidopsis* requires the simultaneous involvement of both the synthetic enzymes (i.e. SS and SBE) and the degradation enzymes (i.e. DBE, AMY) to support starch synthesis. The degradation enzymes can be regarded as responsible for CLD (structural) editing in order for the CLD to attain a steady state. (2) In the wild-type *Arabidopsis*, ISA-type DBEs are the preferred enzymes to carry out the structural editing. This is inferred from the finding that when the DBE is involved, the rate of stead-state attainment is insensitive to the other structural editing enzymes, AMY and BAM. It appears that hydrolysis of *α*-(1→6) linkages by DBEs is an effective structural editing mechanism for supporting crystallization for starch synthesis. This is consistent with the fact that a large number of DBE mutants result in reduced or abolished starch synthesis and cause the accumulation of water-soluble glucan (phytoglycogen). Mutations in other types of structural editing enzymes do not exhibit such striking phenotypes. For example, there was no report of phytoglycogen in the AMY [13] or BAM [16] mutants. This may be because phtoglycogen was not accumulated in significant amounts. (3) The values of the eigenvalues for the ISA-type DBE mutant with AMY^+^/BAM^+^ are less negative compared to those in the wild-type plant (see above). This indicates the relatively rate of steady-state CLD attainment in the ISA-type DBE mutant is slow compared to the wild-type plant. This is the first indication of the possibility that crystallization of glucans is slower in DBE mutants, as proposed by Myers *et al*. [1] and Delatte *et al*. [8].

Model simulations showed in the case of a complete absence of all DBEs, the involvement of either AMY or BAM or both is able to satisfy the zero-eigenvalue condition for the attainment of a steady-state CLD. This explains why in the *Arabidopsis* mutant lacking all DBEs (ISA1, ISA2, ISA3 and PUL) and AMY (a quintuple mutant), starch granule synthesis is still observed [11]. The CLD under this circumstance is significantly enriched in short chains, especially DP 3, followed by DP 2. Our simulation shows a complete absence of DBE with only SS, SBE and BAM yield a CLD with the same characteristics as experimentally observed [11]. This implies BAM becomes the main enzyme contributing to the attainment of a steady-state CLD in the quintuple mutant.

The model fitting shows the involvement of BAM is responsible for the elevated Features A′ and A in the ISA-type DBE mutant. The small differences at Feature A between *Atisa1-1* CLD and simulated CLD with AMY^+^/BAM^+^ (Figure 1H) suggests also that, besides BAM, the glucans are acted on by additional types of biosynthetic enzymes not normally seen as partaking in transient starch synthesis. Phytoglycogen CLD is also enriched in Features A′ and A chains [8], [34]. On this basis, we propose that BAM is one of the major enzymes contributing to the formation of phytoglycogen in *Arabidopsis* ISA-type DBE mutant.

## Conclusions

Our transient starch CLD model shows that, in addition to the core biosynthetic enzymes, AMY is also involved in the trimming of the pre-amylopectin for starch synthesis in *Arabidopsis* leaves. In the ISA-type DBE mutant, both AMY and BAM are involved. AMY, and to a lesser extent BAM, are responsible for the presence of Feature A′ in *Arabidopsis* leaf starch CLD. Our model suggests that neither AMY or BAM regulate the rate of steady-state CLD attainment in wild-type *Arabidopsis*; this instead is controlled by the ISA-type DBE. BAM is detrimental to the rate of steady-state CLD attainment as it attacks non-reducing ends and competes with the ISA-type DBE for trimming of pre-amylopectin. The model shows it is possible for AMY and BAM to support starch synthesis in mutants lacking all DBEs. DBEs are not the only enzymes that can facilitate steady-state CLD attainment for starch synthesis. These results thus provide new perspectives on the roles of AMY and BAM in transient starch synthesis.

## Supporting Information

Figure S1 ***Arabidopsis*** **transient starch CLDs.**
(DOCX)

Figure S2 **The full list of eigenvalues as mentioned in** **Figure 3** **.**
(DOCX)
