# Supplementary material for: New Perspectives on the Role of α- and β-Amylases in Transient Starch Synthesis
Source: PLoS One. 2014 Jun 27;9(6):e100498. doi: 10.1371/journal.pone.0100498 (PMC4074105; doi:10.1371/journal.pone.0100498)
Supplement: Figure S1 — Arabidopsis transient starch CLDs. (DOCX) [file pone.0100498.s001.docx]

| 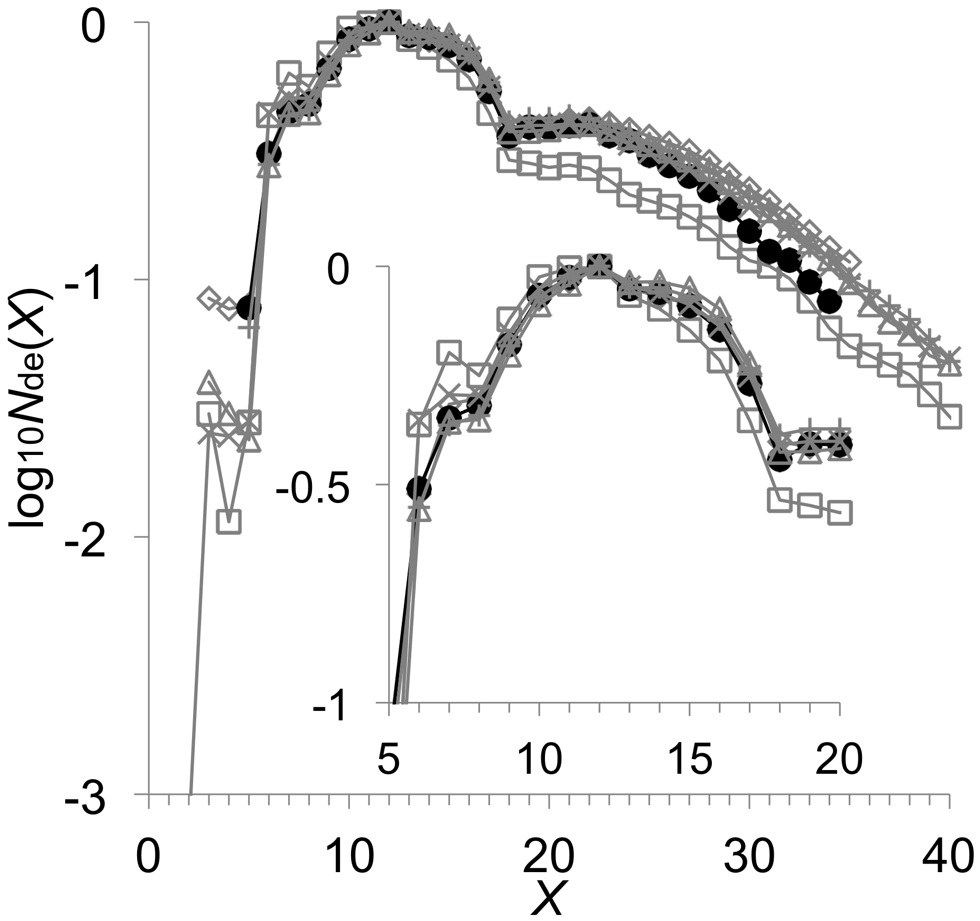 |
| --- |
| Figure S1. *Arabidopsis* transient starch CLDs. The insert is an enlarged view of the first 20 *X*. *X* is the symbol for degree of polymerization. Sources: [[1](#_ENREF_1)] (unfilled squares); [[2](#_ENREF_2)] (unfilled triangles); [[3](#_ENREF_3)] (unfilled diamonds); [[4](#_ENREF_4)] (filled circles); [[5](#_ENREF_5)] (crosses); [[6](#_ENREF_6)] (upright crosses). |

# References

1. Delatte T, Trevisan M, Parker ML, Zeeman SC (2005) Arabidopsis mutants Atisa1 and Atisa2 have identical phenotypes and lack the same multimeric isoamylase, which influences the branch point distribution of amylopectin during starch synthesis. Plant Journal 41: 815-830.

2. Yu TS, Kofler H, Hausler RE, Hille D, Flugge UI, et al. (2001) The Arabidopsis sex1 mutant is defective in the R1 protein, a general regulator of starch degradation in plants, and not in the chloroplast hexose transporter. Plant Cell 13: 1907-1918.

3. Wattebled F, Planchot V, Dong Y, Szydlowski N, Pontoire B, et al. (2008) Further Evidence for the Mandatory Nature of Polysaccharide Debranching for the Aggregation of Semicrystalline Starch and for Overlapping Functions of Debranching Enzymes in Arabidopsis Leaves. Plant Physiology 148: 1309-1323.

4. Wattebled F, Dong Y, Dumez S, Delvalle D, Planchot R, et al. (2005) Mutants of Arabidopsis lacking a chloroplastic isoamylase accumulate phytoglycogen and an abnormal form of amylopectin. Plant Physiology 138: 184-195.

5. Streb S, Eicke S, Zeeman SC (2012) The Simultaneous Abolition of Three Starch Hydrolases Blocks Transient Starch Breakdown in Arabidopsis. Journal of Biological Chemistry 287: 41745-41756.

6. Szydlowski N, Ragel P, Hennen-Bierwagen TA, Planchot V, Myers AM, et al. (2011) Integrated functions among multiple starch synthases determine both amylopectin chain length and branch linkage location in Arabidopsis leaf starch. Journal of Experimental Botany 62: 4547-4559.
