# Supplementary figures and images for: New Perspectives on the Role of α- and β-Amylases in Transient Starch Synthesis
Source: PLoS One. 2014 Jun 27;9(6):e100498. doi: 10.1371/journal.pone.0100498 (PMC4074105; doi:10.1371/journal.pone.0100498)

| 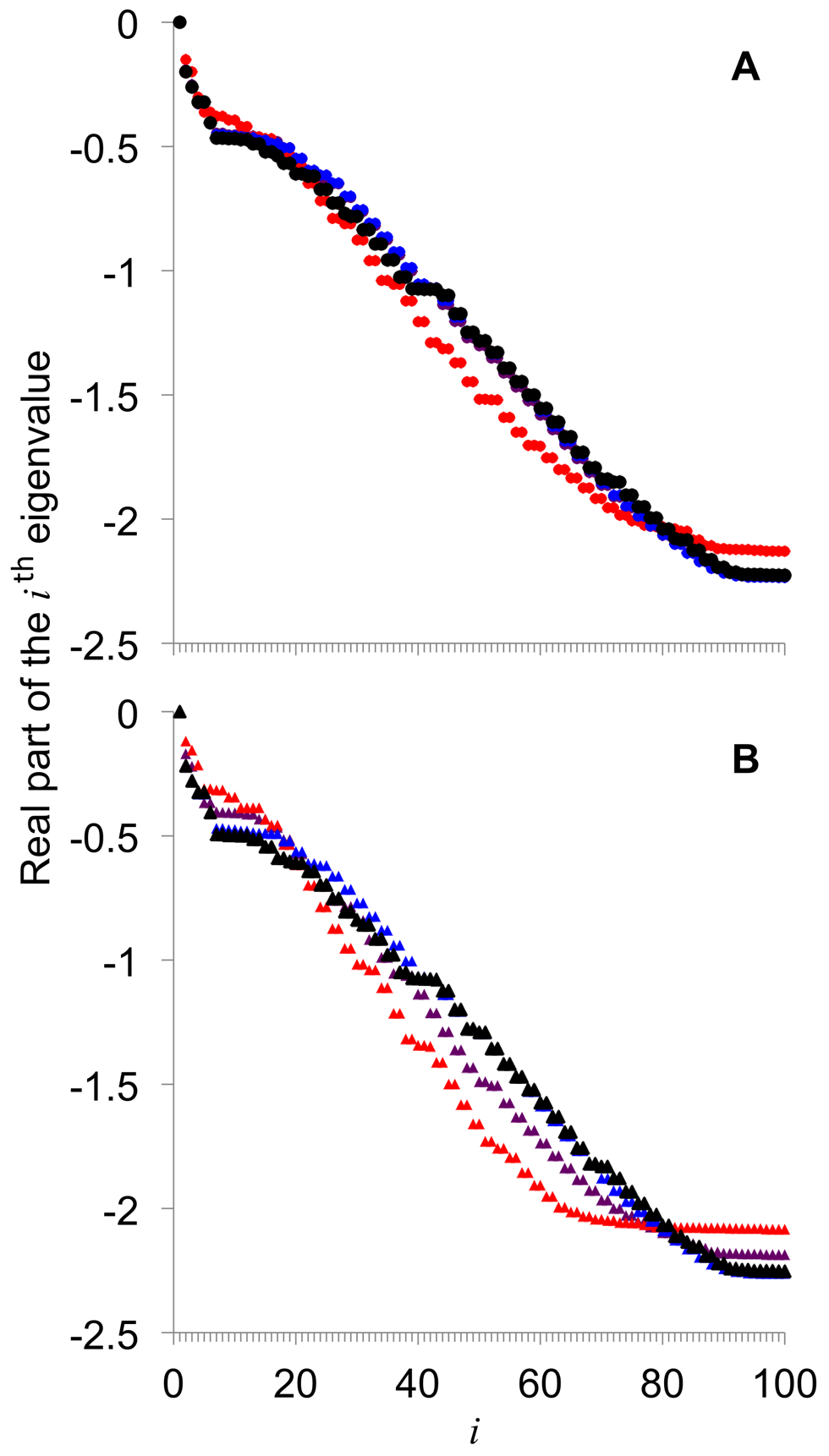 |
| --- |
| Figure S2. The full list of eigenvalues as mentioned in Figure 3. |

Supplement: Figure S2 — The full list of eigenvalues as mentioned in Figure 3 . (DOCX) [file pone.0100498.s002.docx]
